# Population-based incidence and serotype distribution of invasive pneumococcal disease prior to introduction of conjugate pneumococcal vaccine in Bangladesh

**DOI:** 10.1371/journal.pone.0228799

**Published:** 2020-02-13

**Authors:** Abdullah H. Baqui, Eric D. McCollum, Arif Mahmud, Arunangshu Roy, Nabidul H. Chowdhury, Iftekhar Rafiqullah, Syed Jafar Raza Rizvi, Nazma Begum, Dipak K. Mitra, Rasheda Khanam, Meagan Harrison, Salahuddin Ahmed, Md Hasanuzzaman, Hafizur Rahman, Maksuda Islam, Zabed B. Ahmed, Md Abdul Quaiyum, Alain Koffi, Nicole Simmons, William Checkley, Lawrence H. Moulton, Mathuram Santosham, Samir K. Saha, for the Projahnmo Study Group in Bangladesh

**Affiliations:** 1 Department of International Health, Johns Hopkins University Bloomberg School of Public Health, Baltimore, Maryland, United States of America; 2 Department of Pediatrics, Eudowood Division of Pediatric Respiratory Sciences, Johns Hopkins University School of Medicine, Baltimore, Maryland, United States of America; 3 Projahnmo Research Foundation, Dhaka, Bangladesh; 4 North South University, Dhaka, Bangladesh; 5 Child Health Research Foundation, Dhaka, Bangladesh; 6 International Centre for Diarrhoeal Disease Research, Bangladesh, Dhaka, Bangladesh; 7 Division of Pulmonary and Critical Care, Johns Hopkins University School of Medicine, Baltimore, Maryland, United States of America; University of South Dakota, UNITED STATES

## Abstract

**Background:**

Bangladesh introduced the 10-valent pneumococcal conjugate vaccine (PCV-10) in 2015. We measured population-based incidence of invasive pneumococcal disease (IPD) prior to introduction of PCV-10 to provide a benchmark against which the impact of PCV-10 can be assessed.

**Methods:**

We conducted population, facility and laboratory-based surveillance in children 0–59 months of age in three rural sub-districts of Sylhet district of Bangladesh from January 2014 to June 2015. All children received two-monthly home visits with one week recall for morbidity and care seeking. Children attending the three Upazilla Health Complexes (UHC, sub-district hospitals) in the surveillance area were screened for suspected IPD. Blood samples were collected from suspected IPD cases for culture and additionally, cerebrospinal fluid (CSF) was collected from suspected meningitis cases for culture and molecular testing. Pneumococcal isolates were serotyped by Quellung. Serotyping of cases detected by molecular testing was done by sequential multiplex polymerase chain reaction.

**Results:**

Children under surveillance contributed to 126,657 child years of observations. Sixty-three thousand three hundred eighty-four illness episodes were assessed in the UHCs. Blood specimens were collected from 8,668 suspected IPD cases and CSF from 177 suspected meningitis cases. *Streptococcus pneumoniae* was isolated from 46 cases; 32 (70%) were vaccine serotype. The population-based incidence of IPD was 36.3/100,000 child years of observations. About 80% of the cases occurred in children below two years of age.

**Discussion:**

IPD was common in rural Bangladesh suggesting the potential benefit of an effective vaccine. Measurement of the burden of IPD requires multiple surveillance modalities.

## Introduction

*Streptococcus pneumoniae* is a leading cause of bacterial pneumonia, meningitis, and sepsis accounting for an estimated 826,000 deaths globally each year in children 1–59 months of age [1]. Two-third of these deaths occur in ten countries with the highest numbers of pneumococcal cases including Bangladesh [1]. Bangladesh alone experiences an estimated 21,000 child deaths annually from pneumococcal diseases [1]. The World Health Organization (WHO) recommends inclusion of the pneumococcal conjugate vaccine (PCV) in childhood immunization programs world-wide, especially in countries with under-5-mortality of >50/1000 live births [2]. Several low- and middle-income (LMICs) countries have introduced the vaccine and documented substantial benefit from the introduction of PCV [3–8]. An efficacy study in the Gambia demonstrated a 16% reduction in all-cause child mortality attributable to PCV [9]. In 2013, the Government of Bangladesh decided to introduce 10-valent PCV (PCV-10) beginning in early 2015 in its national immunization program with co-financing from **t**he Global Alliance for Vaccines and Immunizations (GAVI). Given Bangladesh’s limited pneumococcal disease burden and serotype data, it was important to carefully assess the impact of national introduction of PCV-10. Pneumococcus has >90 serotypes. The current 10- and 13-valent vaccines were developed based on prevalent serotypes that cause most of the disease in the high-income countries preventing >80% of the invasive pneumococcal diseases (IPD) in those countries. The preventable fraction may be lower in LMICs, such as Bangladesh [10, 11]. Some countries have observed serotype replacement of pneumococcus after introduction of PCV [12–15]. To document the impact of PCV-10, it is important to document the disease burden and the serotype distribution of the pneumococcus prior to the introduction of the vaccine.

We initiated a population and facility-based surveillance on January 1, 2014 to estimate the incidence and serotype distribution of IPD prior to introduction of PCV-10 to provide a benchmark against which the impact of PCV-10 can be assessed. This paper presents the data on population-based incidence, seasonality and serotype distribution of IPD in one district of Bangladesh prior to introduction of PCV-10. We also highlight the challenges of estimating the population-based incidence of IPD in settings characterized by a pluralistic health system in which many households use a range of public, private, and informal health care providers, many of whom are not controlled by national health authorities.

## Methods

The detailed methodology of this impact assessment study was published [16]. Briefly, the study was conducted in an established field research site in Sylhet, Bangladesh. The site was established in 2001 by the Projahnmo Study Group which is a research partnership of the Johns Hopkins University in USA, the Bangladesh Ministry of Health and Family Welfare (MOHFW) and Bangladeshi non-governmental organizations (NGOs). The site encompasses three *Upazillas* (sub-districts) of Sylhet district of Bangladesh (Zakiganj, Kanaighat and Beanibazar) with an estimated population of 770,000 and an annual birth cohort of about 20,000. All households, women and children in the study area have unique current and permanent identifiers, which allow individual tracking and longitudinal follow-up. The study was conducted among all children 0–59 months of age residing in study area.

We established community- and facility-based surveillance in all *Upazilla* Health Complexes (UHCs, government sub-district hospitals) in the entire study area for detection of suspected IPD including meningitis cases and collection and transport of specimens.

The protocol was approved by the institutional review board of the International Centre for Diarrhoeal Disease Research, Bangladesh (icddr,b) in Dhaka, Bangladesh (PR-13095) and Johns Hopkins Bloomberg School of Public Health in Baltimore, Maryland, USA (IRB00005421).

### Community-based surveillance

Trained female community health workers (CHW) who were residents of the community and had at least a 10th grade education, each were assigned to a population of about 10,000. Each CHW visited all households in her area (average 2,000 households) once every two months to update the population data by recording all new pregnancies, births, deaths, marriages and movements. Background data on age, parity, literacy, prior obstetric history and socio-economic status of all women in the study area were collected. CHWs provided information to all women of child-bearing age regarding care during pregnancy, delivery, postpartum period and for childhood illnesses during the two-monthly home visits. Families were encouraged to notify CHWs about all births as soon as possible using a cell phone-based birth notification system and to seek care from study designated hospitals for any illnesses in newborns and children. During surveillance visits, CHWs collected data on vital status, illness history and care seeking for all 0–59 months old children until they were 60 months of age or died or migrated out of the study area. To minimize recall lapse, the recall period for illnesses and care seeking in each visit was one week. Mothers and family members were educated during two-monthly home visits on signs of sepsis, meningitis and pneumonia in children and were further encouraged to visit the designated study hospitals for clinical evaluation, management, and enrollment of suspected IPD cases in the study.

Since care seeking from study designated hospitals was low from remote villages, the CHWs recruited and supervised local resident female village health workers (VHWs) in those villages. A VHW was recruited for a village of about 1,000 people. Since many of the VHWs were illiterate, they were provided with thermometers marked at a cutoff level of 101°F and trained on their use. They were also trained to recognize signs of pneumonia and meningitis. In addition to CHW’s 2-monthly visits, the VHWs visited and screened children of consented parents weekly for fever, respiratory problems and danger signs including convulsions, and referred sick children to one of the study hospitals. The VHWs received nominal monthly stipends and financial incentives for successful referrals of eligible children with high fever, clinical signs of pneumonia and meningitis. VHWs did not collect any study data but facilitated case detection and referral from surveillance population to the study hospitals. In case of refusal of referral advice by families, the VHWs called the CHWs for further facilitation of the referral process [16].

The study area had many different types of health care providers including informal providers. In addition to promoting care seeking from sub-district hospitals, we developed a network with the first-level facilities and private health care providers in the formal and informal sectors within the study area and encouraged them to refer sick children with suspected sepsis, pneumonia or meningitis to the designated study hospitals. We also included Sylhet Medical College and other private tertiary level referral hospitals in Sylhet City in this network, which were frequent referral sites for children with signs of meningitis in the study area. We mapped the first-level clinics and private health care providers in the study area including government Family Welfare Centers (FWCs), Community Clinics, NGO-supported clinics, village doctors, and drug sellers. All these providers were given orientation on the study and were requested to refer eligible cases to study designated hospitals. The orientation included use of standardized criteria for diagnosing suspected pneumonia and meningitis cases and instruction on keeping a list of cases by village. Study physicians (doctors with MBBS degrees from Bangladeshi medical colleges) periodically visited these providers and facilities to assess the quality of diagnoses made and reinforced adherence to the case definitions and protocols for referral of all suspected cases to designated health facilities. We identified informal providers who saw a particularly high volume of sick children <5 years of age and obtained the providers’ consent to station mobile teams of trained health workers at their offices.

### Hospital surveillance and processing of specimens

Upon presentation at the participating study hospitals, a research assistant requested parents of all <5-year-old children to provide consent for screening for suspected IPD (clinical pneumonia, meningitis, or high fever) and for collection of basic demographic and clinical information. Suspected IPD cases were identified by study physicians at the study hospitals and mobile team members in the community. After obtaining written informed consent from parents, detailed data on demographic characteristics, clinical assessment, and history of medication use were recorded in a case report form (CRF). A blood sample (~3 ml) was collected from each clinically eligible child whose parents provided consent and who did not receive antibiotic before seeking care. Half of the blood was directly inoculated into a BACTEC (Becton Dickinson Diagnostic Instrument Systems, Sparks, MD, USA) pediatric blood culture bottle and kept at room temperature until transportation to the Sylhet laboratory, where it was processed in a BACTEC machine. The culture bottles that showed signs of bacterial growth were sub-cultured. The other half of the blood specimen was transported to the Sylhet laboratory maintaining cold chain for molecular testing. Lumbar puncture was performed for all consented hospitalized cases with signs of meningitis. About 2 ml of CSF was collected for immediate plating on chocolate, blood and MacConkey agar media and a portion of CSF was saved for cytology and measurement of biochemical parameters. The remaining CSF specimen was stored for molecular testing. All blood specimens were transported to the study laboratory in Sylhet within 8 hours. All CSF specimens were transported immediately for identification and real-time assessment of biological parameters. Preliminarily identified pneumococcal isolates were transported to a central laboratory at Dhaka Shishu Hospital in Dhaka for reconfirmation and serotyping. All molecular testing was performed in the central lab using real-time polymerase chain reaction (PCR) techniques. The study doctors recorded data on treatment given, status at discharge and laboratory findings in the CRF. We identified IPD cases using a pre-determined criterion that took in to account the clinical findings, culture and molecular test results [11]. IPD was defined if *streptococcus pneumonia* was isolated from blood or CSF. All sick children received treatment based on the physicians’ clinical diagnosis. For outpatients, a written report on blood culture results was provided to parents at the next routine household visit after the results became available.

For meningitis cases treated in non-study hospitals, a trained study physician traveled to visit patients in that hospital, obtained parental informed consent, assessed the patients for suspected meningitis, and performed lumbar puncture if appropriate. The cerebrospinal fluid (CSF) specimen was transported to the study laboratory using the procedures described above. All reports were promptly shared with the treating hospital.

We established a quality control program to ensure the quality of screening to optimize enrollment of eligible cases and of blood specimen collection to avoid specimen contamination. Periodic and need-based refresher trainings were organized for all study staff.

### Analysis

A child contributed to person-time for the study from the time when the child was registered (entry date) until the child became 60 months old or died, or moved out of the study area (exit date). For analysis of community morbidity and care-seeking data, we used weeks of observations as the denominator counting the number of children who were present during the 2-monthly home visits. To calculate hospital visit rate and IPD rates, we used age- and sex-specific child years of observations.

## Results

**Fig 1:**
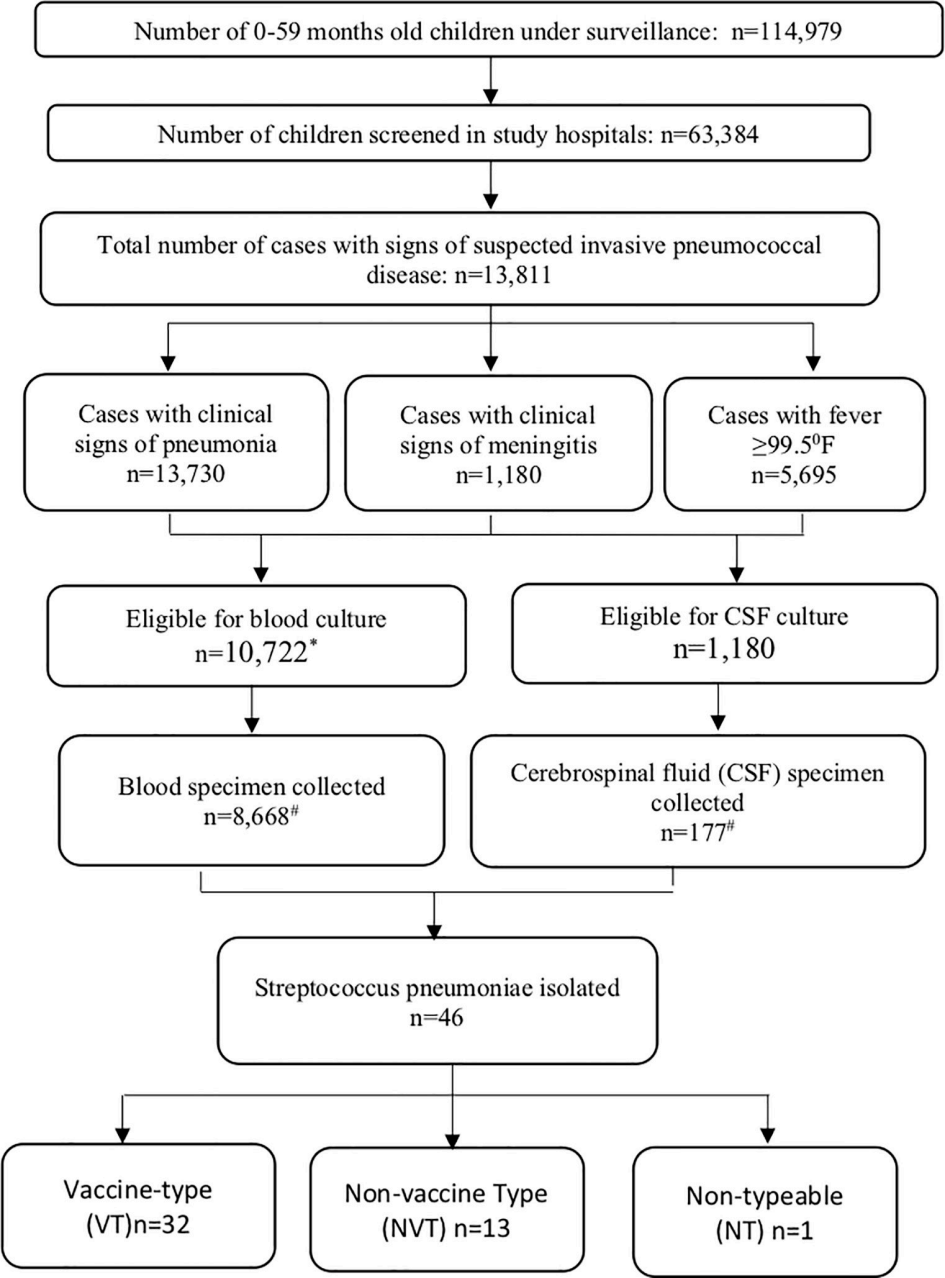
Study flow chart, January 2014—June 2015.

Between January 2014 and June 2015, a total of 114,979 children were under surveillance (Fig 1). The total child years of observations was 126,657. Sixty-three thousand three hundred eighty-four illness episodes were assessed in the out-patient clinics of the UHCs. Of the 13,811 episodes with signs of suspected IPD, 13,730 had signs of pneumonia, 1,180 had signs of meningitis and 5,695 had high fever. Blood specimens were collected from 8,668 episodes and cerebrospinal fluid was collected from 177 episodes. *Streptococcus pneumoniae* was isolated from 46 cases. Of these 32 were vaccine serotype, 13 were non-vaccine type and one was non typeable (Fig 1).

With two-monthly home visits and one week of recall for morbidity and care seeking, the children contributed to 499,746 child weeks of observation. Overall, the prevalence of pneumonia was 2.0%, meningitis was 0.05%, and isolated high fever was 12.1%. The prevalence of pneumonia was highest in children 3–5 months (3.5%), followed by 6–11 months (3.3%) old. The prevalence of suspected meningitis was highest in children 6–23 months (0.07%) and prevalence of isolated high fever was highest in children 6–11 months (16.1%). The prevalence of all three conditions was lowest in 48–59 months old children (Table 1).

**Table 1:** Weekly prevalence of suspected pneumonia, meningitis and isolated fever based on two-monthly home visit surveillance, stratified by age, January 2014-June 2015, Sylhet, Bangladesh.

Age in months	Number of children who were present during visit (N)	Number of suspected Pneumonia cases N (%)	Number of suspected Meningitis cases N (%)	Number of isolated fever Cases N (%)	Number of suspected IPD cases N (%)
0–2	19,608	399 (2.0)	11 (0.06)	1,911 (9.7)	2,321 (11.8)
3–5	23,074	810 (3.5)	7 (0.03)	3,349 (14.5)	4,166 (18.1)
6–11	48,353	1,582 (3.3)	35 (0.07)	7,781 (16.1)	9,398 (19.4)
12–23	104,995	2,557 (2.4)	78 (0.07)	14,646 (13.9)	17,281 (16.5)
24–35	108,152	2,059 (1.9)	45 (0.04)	13,103 (12.1)	15,207 (14.1)
36–47	101,332	1,409 (1.4)	27 (0.03)	10,736 (10.6)	12,172 (12)
48–59	94,232	1,034 (1.1)	24 (0.03)	8,774 (9.3)	9,832 (10.4)
Total	499,746	9,850 (2.0)	227 (0.05)	60,300 (12.1)	70,377 (14.1)

Only 7.7% of the community reported cases with suspected IPD sought care from the study designated hospitals, the UHCs. The main providers were village doctors (42.6%) and about a third of the reported cases (30.7%) did not seek any care. The proportions of pneumonia, suspected meningitis and high fever cases that sought care from the sub-district Health Complexes were 12.4%, 18.9% and 6.9%, respectively (Table 2).

**Table 2:** Care-seeking by reported morbidity categories based on two-monthly home visit surveillance, January 2014-June 2015, Sylhet, Bangladesh.

Place of care	Suspected Pneumonia (%)	Suspected Meningitis (%)	Isolated Fever (%)	Suspected IPD (%)
No Care	2,376 (24.1)	37 (16.3)	19,212 (31.9)	21,625 (30.7)
Home	142 (1.4)	5 (2.2)	1,759 (2.9)	1,906 (2.7)
Pharmacy/ Village doctor	4,008 (40.7)	63 (27.8)	25,872 (42.9)	29,943 (42.6)
**Upazilla Health Complex**	1,216 (12.4)	43 (18.9)	4,131 (6.9)	5,390 (7.7)
Medical College Hospital	56 (0.6)	7 (3.1)	75 (0.1)	138 (0.2)
Private (office, clinic, hospital)	1,443 (14.7)	62 (27.3)	5,533 (9.2)	7,038 (10)
Satellite/Community Clinic/FWC	399 (4.1)	3 (1.3)	2,027 (3.4)	2,429 (3.5)
Don't know/ Other	210 (2.1)	7 (3.1)	1,691 (2.8)	1,908 (2.7)
Total	9,850	227	60,300	70,377

Of the 13,811 clinically eligible cases for blood culture, 3,089 (22.4%) were excluded because they received antibiotics prior to seeking care from the UHCs. Of the 10,722 enrollable cases, 1,948 (18.2%) did not provide consent and blood could not be collected from another 106 (<1.0%) cases either because the child was very sick, or the phlebotomist could not collect blood. Of the 1,180 clinically eligible cases for lumbar puncture, 5 cases were excluded because they were enrolled as potential IPD cases in the preceding 7 days. Of the 1,175 enrollable cases, 970 cases did not consent, and CSF could not be collected from another 28 enrollable cases for other reasons (attempt failure, not in condition to collect CSF) (Table 3).

**Table 3:** Age-specific number of hospital visits, eligible cases and blood and CSF specimen collected among children 0–59 months of age, January 2014-June 2015, Sylhet, Bangladesh.

Age in months	Screened		Blood culture		CSF culture		
		Clinically eligible	Enrollable^a^	Blood collected^b^	Clinically eligible	Enrollable^c^	CSF collected^d^
0–2	5,814	1,189	903	727 (80.5%)	113	111	6 (5.4%)
3–5	6,437	1,585	1,193	944 (79.1%)	123	122	10 (8.2%)
6–11	11,580	2,829	2,139	1,680 (78.5%)	241	241	37 (15.4%)
12–23	16,299	3,730	2,872	2,302 (80.2%)	349	349	53 (15.2%)
24–35	11,743	2,492	2,014	1,672 (83.0%)	193	193	44 (22.8%)
36–47	7,083	1,289	1,037	865 (83.4%)	107	106	23 (21.7%)
48–59	4,428	697	564	478 (84.8%)	54	53	4 (7.6%)
Total	63,384	13,811	10,722	8,668 (80.8%)	1,180	1,175	177 (15.1%)

^a^ 3,089 cases were eligible but were excluded due to prior antibiotic use or enrollment as a potential IPD case in the preceding 7 days

^b^1,948 were enrollable but did not provide consent; 106 cases were enrolled but had no blood collected due to other reasons (attempt failure, not in condition to collect blood)

^c^ 5 cases were eligible but were excluded due to enrollment as a potential IPD case in the preceding 7 days.

^d^ 970 enrollable cases did not consent; CSF not collected from another 28 enrollable cases for other reasons (attempt failure, not in condition to collect CSF)

**Fig 2:**
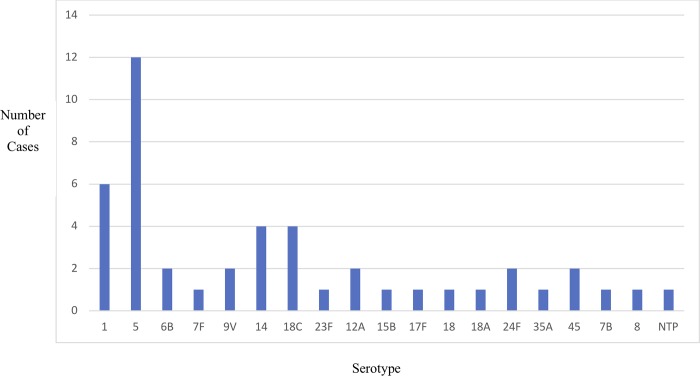
Serotype distribution of pneumococcal isolates prior to introduction of PCV-10, Sylhet, Bangladesh.

We isolated pneumococcus from 46 cases, 44 from blood specimens and 2 from CSF. Thirty-two isolates (69.6%) were vaccine type, 13 (28.3%) were non-vaccine type and one was non-typable. The most common serotype was serotype 5 (Fig 2).

The majority of the IPD cases were in infants (28/46, 60.9%) below 12 months of age and 78.3% were in the first two years of life (Table 4).

**Table 4:** Number of PCV-10 type and non-vaccine type *Streptococcus pneumoniae* isolated from blood and CSF samples by age among children 0–59 months of age, January 2014-June 2015, Sylhet, Bangladesh.

Age (month)	Hospital visits	Blood cultures perform-ed	*Streptococcus Pneumoniae* isolated from blood specimens				CSF cultures perform-ed	*Streptococcus Pneumoniae* isolated from CSF specimens on culture or molecular test				All IPD-positive culture results		
	(n)	(n)	VT	NVT	NT	Total*	(n)	VT	NVT	Total*	VT	NVT	NT	Total*
0–2	5,814	727	3	1	0	4	6	1	0	1	4	1	0	5
3–5	6,437	944	2	3	1	6	10	0	1	1	2	4	1	7
6–11	11,580	1,680	10	6	0	16	37	0	0	0	10	6	0	16
12–23	16,299	2,302	6	2	0	8	52	0	0	0	6	2	0	8
24–35	11,743	1,672	5	0	0	5	44	0	0	0	5	0	0	5
36–47	7,083	864	2	0	0	2	23	0	0	0	2	0	0	2
48–59	4,428	477	3	0	0	3	4	0	0	0	3	0	0	3
**Total**	63,384	8,666**	31	12	1	44	176**	1	1	2	32	13	1	46

* Includes non-typable/unknown

**There were two blood samples and 1 CSF sample with no report.

Overall IPD rate was 36.3/100,000 child years of observation, 38.8/100,000 in males and 33.7/100,000 in female children (Table 5). IPD rate was highest in infants 6–11 months of age (124.5/100,000; 95% Confidence Interval: 71.2–202.2), followed by infants 3–5 months of age (101.5/100,000; 95% Confidence Interval: 40.8–209.2).

**Table 5:** Age and sex-specific IPD case rate by year in active surveillance area per 100,000 child-years observed.

Age in Month	Baseline (Jan 2014-Jun 2015)								
	Child year observed			# of IPD cases			IPD case rate per 100,000 child years		
	Male	Female	Total	Male	Female	Total	Male	Female	Total
0–2 Months	3,263	3,210	6,472	5	0	5	153.2 (49.8–357.8)	0.0 (0–0)	77.3 (25.1–180.2) *
3–5 Months	3,459	3,436	6,895	1	6	7	28.9 (0.7–161.1)	174.6 (64.1–380.1)	101.5 (40.8–209.2)
6–11 Months	6,491	6,360	12,851	9	7	16	138.7 (63.4–263.2)	110.1 (44.3–226.8)	124.5 (71.2–202.2)
12–23 Months	13,630	13,365	26,996	2	6	8	14.7 (1.8–53.0)	44.9 (16.5–97.8)	29.6 (12.8–58.4)
24–35 Months	13,690	13,269	26,960	4	1	5	29.2 (8.0–74.8)	7.5 (0.2–42.0)	18.6 (6.0–43.3)
36–47 Months	12,408	11,813	24,221	1	1	2	8.1 (0.2–45.0)	8.5 (0.2–47.2)	8.3 (1.0–29.8)
48–59 Months	11,480	10,782	22,262	3	0	3	26.1 (5.4–76.4)	0.0 (0.0–34.2)	13.5 (2.8–39.4)
Total	64,422	62,235	126,657	25	21	46	38.8 (25.1–57.3)	33.7 (20.9–51.6)	36.3 (26.6–48.4)

*95% confidence interval

**Fig 3:**
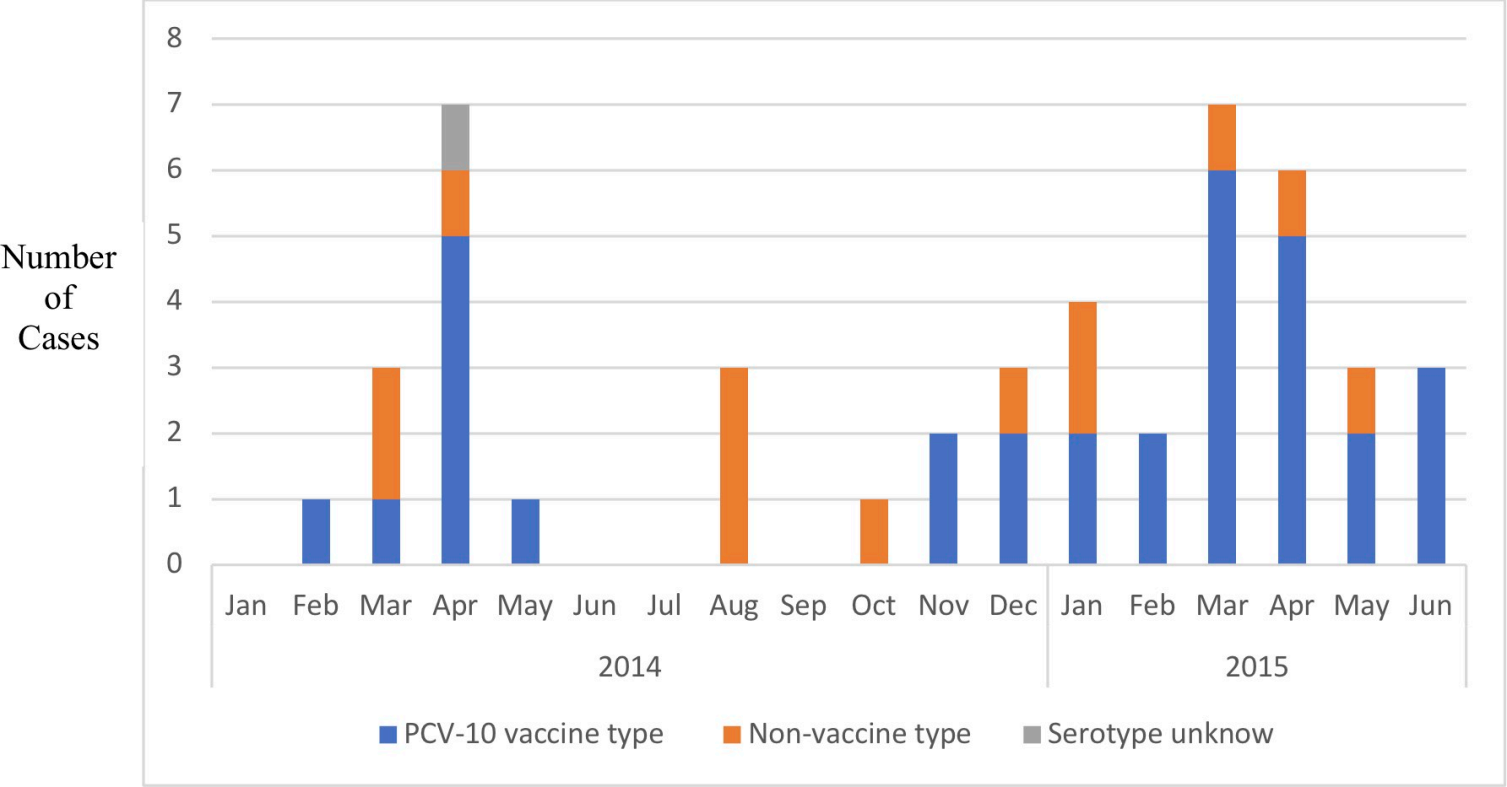
Serotype distribution and seasonality of pneumococcal isolates.

There was a seasonal pattern in the IPD cases; with peaks in March-April periods (Fig 3).

## Discussion

The population-based incidence of IPD in our study area was 36.3/100,000 child years of observations. About 70% of the cases were PCV-10 type and about 80% of the cases occurred in children below two years of age. The IPD rate was 106.8/100,000 child years in the first year of life, 67.7/100,000 child years in children below two years of age and only 10.9/100,00 children in the 2–4 years age group.

Most of the earlier data on IPD, particularly from South Asia, were from health facility or laboratory-based surveillance [17]. Our study provides valuable data on population-based incidence of IPD and insights on the challenges of establishing population-based IPD rates in settings characterized by pluralistic health systems with a large number of informal healthcare providers. We observed an IPD rate that was on the lower end of the range of rates reported. The reported rates vary widely ranging from 15 cases per 100,000 child-years in Mali [18] to 34 cases per 100,000 child years in Chile [19], 86 cases per 100,000 child years in an earlier study in Bangladesh [20], 171 cases per 100,000 child years in The Gambia [21], and 426 cases per 100,000 child years in Kenya [22]. A study from Mozambique conducted between 2001–2012 reported IPD incidence rates of 479, 390, and 107 episodes per 100,000 children-years at risk among children <12, 12–23 and 24–59 months old, respectively [23]. Our rates are similar to the incidence rates reported from South Africa in the pre PCV era: 87 per 100,000 and 14/100,000 in children <1 and 1–4 years of age respectively [24].

The reasons for these large variations in IPD rates are not entirely clear. There were differences in surveillance methods, case definitions, eligibility criteria, laboratory methods, and duration of surveillance across study sites. Some of the differences were likely due to differences in methodologies, but some are likely to be true population differences. A study from South Africa reported about a 20-fold higher risk of IPD in the HIV positive population [24].

Bangladesh has now introduced the PCV-10. We identified 19 different serotypes, and eight of them are PCV-10 type. Our data suggest that this vaccine will prevent about 70% of the pneumococcal diseases in Bangladesh. However, because several of the serotypes we isolated are not included in PCV-10, a substantial number of IPD cases would continue to occur in Bangladesh. To provide the greatest public health benefits, a vaccine that provides species-wide protection or includes a greater number of serotypes is needed.

### Limitations and challenges

Our observed IPD rate was an underestimation of the true burden for several reasons. First, the IPD data prior to the introduction of PCV-10 was limited to 18 months duration. Our efforts to establish the surveillance and capture the majority of the cases in a large surveillance population was inadequate, particularly in the beginning of the study. It took several months to establish the community surveillance and referral system. Therefore, the effective surveillance likely occurred for just one year from July 2014 to June 2015.

Second, less than 10% of the cases reported during the two-monthly home visits sought care from the study designated hospitals. Community cases can’t be directly compared with the clinical IPD cases ascertained in the hospitals for blood or CSF culture because we used a more sensitive criteria to ascertain cases in the community to maximize referral. Therefore, the actual proportion of potential IPD cases in the community that sought care from study hospitals was presumably much higher than 10% but it is difficult to quantify. We observed that distance was a major barrier to care seeking by clinical IPD cases. The hospital visit rate for suspected IPD was about 20 cases per 100 child years of observation for children who lived within 5 Km of the study designated hospitals. The rate steadily declined to about 1–2 cases per 100 child years of observation for children living 25–30 KM away from the study hospitals. If distance was not a barrier, the predicted number of hospital visits by potential IPD cases would have been about double. If we assume that the isolation rate of pneumococcus would have remained the same with this increased care seeking rate from the study hospitals, the IPD rate would have been very similar to an earlier study from Bangladesh (86 cases per 100,000 child years) [20].

A factor related to the low rates of care seeking is that the main care providers in this study area were untrained village doctors. The preponderance of care seeking for childhood illnesses from village doctors is common all over Bangladesh and in many other countries of South Asia [25]. We enumerated all the village doctors, mapped their locations, and collected data on the volume of service they were providing. The study area had 866 villages and on average there was an untrained village doctor in each village. Only about 68 of these village doctors were providing about half of the care and these doctors were clustered in 7 village markets, hereafter referred to as “high volume providers”. We organized orientation sessions for all the village doctors in our efforts to encourage them to refer pneumonia, meningitis and high fever cases to study hospitals. There were very few referrals from village doctors. We then engaged with the high-volume providers to seek their assistance with referral, but they advised that we create mobile teams for assessments and blood collection in their clusters of practices. Accordingly, we recruited and deployed 9 mobile teams staffed by trained paramedics to work with the high-volume providers, but this strategy was met with limited success. Over a 12-month period we identified 392 cases, collected 308 blood sample, and identified one IPD case.

Additionally, the observed IPD rate was likely to be an underestimate because 22.4% of clinically blood culture eligible cases came to the hospital after receiving antibiotics, and only 15% of clinical meningitis cases provided CSF. Based on limited clinical data it seems that cases that consented to CSF were potentially more severe with higher rates of convulsions, but this could not be adequately assessed. Antibiotic use in this population is characterized by a pluralistic healthcare system with widespread availability of over the counter antibiotics. Finally, hospitalization of meningitis cases and lumbar puncture was not a routine procedure in the surveillance hospitals and therefore, obtaining consent for lumbar puncture was a major challenge.

The study also had several strengths. We established population- and facility-based surveillance in a large population and employed multiple strategies to capture cases. Considering the challenges of time required to transport specimens from collection point to lab, we pre-incubated subculture of all blood culture bottles who were delayed >7 hrs to arrive at lab, to avoid autolysis. We performed PCR of beep positive culture negative cases to rule out or detect pneumococcus, used selective media to minimize missing of pneumococcus among the fast growing contaminated organism, and use realtime PCR to detect pneumococcus from culture negative CSF specimens [26].

We have demonstrated substantial burden of IPD in Bangladesh, particularly in children <2 years of age and recommend sustained use of PCV10. Implementation of PCV has been slow, particularly in South Asia, because of limited local pneumococcal disease burden and serotype data, high cost of the vaccine, and size of the populations [1]. While policy makers in many countries prefer data from their own populations, we observed that establishing population-based pneumococcal disease surveillance to measure IPD burden is not simple and fraught with underestimations of the burden [16]. Based on our own data and other available data, we also recommend that other South Asian countries yet to introduce PCV should consider introduction of PCV in their routine immunization program.
